# Strategic latency with temporal mutualism: a coevolutionary model of the host–varicella-zoster virus relationship

**DOI:** 10.3389/fimmu.2025.1682707

**Published:** 2025-10-02

**Authors:** Dong-Gyun Han

**Affiliations:** National Medical Center, Seoul, Republic of Korea

**Keywords:** varicella-zoster virus, latency, immunosensor, sensory ganglia, temporally partitioned evolutionarily stable strategy (TP-ESS), coevolution, game theory

## Abstract

After primary infection with varicella, varicella-zoster virus (VZV) establishes a latent state that precedes the clinical manifestation of herpes zoster. Growing evidence, however, indicates that latency is not merely quiescent but represents an active immunological adaptation. We propose the *immunosensor hypothesis*, in which VZV latency within sensory ganglia contributes to host immune surveillance while simultaneously ensuring viral persistence. Using a game-theoretical framework, we conceptualize this interaction as a temporally partitioned evolutionarily stable strategy (TP-ESS). In this model, VZV progresses through three sequential phases across the host lifespan: (i) aggressive replication and transmission during primary infection, (ii) immunomodulatory latency during immune competence, and (iii) reactivation during immune decline. Each phase represents a dynamic equilibrium shaped by host immunity and viral life-history trade-offs. The TP-ESS framework integrates viral ecology, innate immunity, and neurovirology into a unified model of latency and reactivation, providing a conceptual basis for epidemiological patterns of herpes zoster and generating testable predictions on immunity-dependent viral behavior and host–virus coadaptation.

## 1 Introduction

As Theodosius Dobzhansky famously remarked, “Nothing in biology makes sense except in the light of evolution” (1). This principle is particularly relevant to understanding the persistence of varicella-zoster virus (VZV) latency within human sensory ganglia.

Over the past decades, numerous proximate mechanisms—the *how* of latency—have been investigated. Rodent studies demonstrated the persistence of viral DNA and restricted transcriptional activity in sensory ganglia. SCID-hu mouse models, using human dorsal root ganglion xenografts, revealed latency-associated transcripts and proteins *in vivo*. In nonhuman primates, simian varicella virus (SVV) faithfully recapitulated primary infection, latency, and reactivation, providing strong parallels to human disease. More recently, human embryonic stem cell–derived neurons have provided tractable *in vitro* systems to probe genome maintenance and transcriptional restriction under controlled conditions (2–6). Collectively, these studies demonstrate that latency is not a state of complete silence but is actively maintained through the persistence of viral genomes within neurons, restricted transcriptional activity, and immune cell infiltration of sensory ganglia.

While these proximate findings have clarified the mechanisms that sustain latency, they cannot by themselves resolve the ultimate question—*why* VZV has evolved to maintain such a finely balanced and apparently costly state (7). In other words, proximate models explain how latency persists, but they do not explain the evolutionary rationale underlying its long-term stability.

Ecological analogies—including commensalism, parasitism, and by extension mutualism—can be used to frame this relationship, yet each has limitations when applied in isolation. Commensalism accounts for the silent persistence of latency but cannot explain the pathogenicity of zoster. Parasitism, by contrast, explains host injury during reactivation but is inconsistent with VZV’s low mutation rate and rare reactivation frequency. Neither framework alone adequately integrates these contrasting aspects of VZV biology. To resolve this gap, we introduce the concept of temporal mutualism, in which latency represents a commensalism-like phase that minimizes host cost, while rare reactivation episodes resemble parasitism at the individual level but reinforce population-level immunity. This framework unifies proximate experimental findings with evolutionary meaning and provides a more coherent model of VZV persistence.

## 2 Strategic latency and temporal mutualism

VZV displays a triphasic life cycle in which each phase represents a distinct viral strategy shaped by host immune surveillance. During primary infection, the virus maximizes replication and transmission; during latency, it persists under continuous immune engagement within sensory ganglia; and during reactivation, it exploits waning or impaired immunity to achieve intergenerational spread. Collectively, these phases constitute a temporally partitioned evolutionarily stable strategy (ESS), in which viral persistence and host survival are balanced through dynamic trade-offs across the lifespan.

### 2.1 Childhood replication and latency establishment

During primary infection, VZV undergoes a highly contagious lytic phase optimized for rapid proliferation and airborne transmission. This typically unfolds in early childhood, when host immunity is still developing and social contact among children is abundant. Following initial replication in mucosal epithelial cells, the virus disseminates via viremia to the skin and sensory nerves, culminating in the vesicular rash of varicella (chickenpox). The incubation period is relatively long, generally 14–21 days, allowing extensive viral amplification before overt symptoms appear (8, 9).

Crucially, VZV latency is established at or shortly after primary infection, irrespective of the age at which varicella is acquired. In most temperate regions this typically occurs in childhood, whereas in tropical regions primary varicella is more frequently delayed until adulthood (10). Herpes zoster, which generally appears decades later, therefore represents the reactivation of a latent infection that was established at the time of primary infection, rather than the *de novo* initiation of latency in adulthood. Following neuroinvasion, viral genomes persist in sensory ganglia immediately after acute infection. Similar to HSV, VZV latency is now recognized as a leaky state rather than a fully quiescent one, characterized by intermittent viral gene expression within sensory ganglia (11). These antigens provide a substrate for continuous immune surveillance, highlighting that latency is not immunologically silent but actively engages host defenses.

### 2.2 Latency maintenance under immune surveillance

Once established, VZV latency is actively maintained by continuous immune surveillance rather than mere quiescence. Immunohistochemical analyses of human dorsal root ganglia have consistently revealed infiltrates of CD3^+^ T cells, including both CD4^+^ helper and CD8^+^ cytotoxic subsets, together with CD56^+^/CD57^+^ NK cells, CD68^+^ macrophages, and B cells in association with latent VZV (2, 3). It should be emphasized, however, that truly virus-free human ganglia are rare, owing to the very high prevalence of latent herpesviruses in adults and the ethical constraints that preclude access to uninfected pediatric ganglia as true controls. Current evidence indicates that VZV latency is established primarily in sensory ganglionic neurons, with only occasional reports of viral detection in satellite glial cells, which may represent rare exceptions rather than the norm (12). Importantly, human post-mortem studies of sensory ganglia in both VZV and HSV have been pivotal in defining the molecular signatures of latency, underscoring the central role of neuropathological specimens in shaping our understanding of herpesvirus biology (11).

Parallel insights have been gained from HSV, where latency is no longer regarded as a state of complete transcriptional silence. Instead, sporadic expression of immediate-early and early genes, and even occasional late proteins, can occur without progression to productive replication. This non-productive yet antigenic activity—termed *animation*—denotes subclinical, intermittent gene expression during latency without productive infection or clinical disease. Animation should therefore be understood not as a lytic strategy but as a functional mode of latency, providing intermittent antigenic signals that sustain T-cell retention within ganglia and reinforce long-term persistence.

Consistent with this paradigm, accumulating evidence indicates that VZV latency is also dynamic rather than strictly silent. VLT transcripts are consistently detected in latently infected human ganglia. Importantly, what was previously interpreted as independent ORF63 expression has since been shown to represent VLT-ORF63 splice variants (11, 13–15). These transcripts can give rise to VLT63 proteins during reactivation but have not been detected in latency. Likewise, earlier reports of IE63 protein expression in latency were later attributed to antibody cross-reactivity with blood group A antigens (16). Thus, current evidence supports VLT RNA as the sole reliable biomarker of VZV latency, while VLT-ORF63 isoforms likely mark the transition toward reactivation.

Simian varicella virus (SVV) models corroborate these findings, showing that latency involves ongoing recruitment of innate effectors, such as monocytes and NK cells, into ganglionic tissue (15, 17). Moreover, primary VZV infection induces durable cell-mediated immunity that plays a pivotal role in preventing reactivation (17, 18). Taken together, latency in VZV should not be conceived as a static state but as a dynamic process in which intermittent viral gene expression sustains continuous immune engagement.

### 2.3 Late-life reactivation enabling intergenerational transmission

In late life or during periods of immunosenescence, this long-maintained equilibrium is disrupted, and latent VZV may reactivate as herpes zoster (shingles). Herpes zoster is generally considered to occur only once in most immunocompetent individuals, with reported recurrence rates of approximately 4.1–6.2% (19). Clinically, this reactivation is characterized by a painful dermatomal rash, and epidemiological data indicate that nearly one-third of adults will experience shingles in their lifetime, with incidence rising sharply after age 50 and reaching up to 11 per 1,000 person-years in those over 65 (20, 21). Importantly, shingles not only reflects waning immune control but also functions as a strategic late-life mechanism for intergenerational transmission, allowing VZV to propagate efficiently within household settings when contact patterns favor viral spread.

The household secondary attack rate of varicella is markedly lower when the primary case is vaccinated (≈15% vs. 71%), indicating that breakthrough infections are about 80% less transmissible within households (22). This underscores how both natural reactivation in older adults and primary infections in vaccinated individuals can contribute to ongoing viral circulation, albeit with differing levels of transmissibility.

Reactivation driven by immunity decline, not age—Although the incidence of zoster peaks in older adults, reactivation can occur in childhood when cell-mediated immune surveillance is impaired (e.g., congenital or iatrogenic immunosuppression, malignancy/chemotherapy, HIV, or in children who experienced varicella early in life). These episodes reflect the same mechanism—failure of immune containment—occurring earlier in life, rather than a distinct pathway (23, 24). Notably, herpes zoster can also occur before adulthood, indicating that either a high viral load at the time of latency establishment or an earlier age of acquisition when immune maturity is incomplete may predispose individuals to earlier reactivation (25). In our ESS framing, late-life reactivation is therefore age-associated but not age-restricted: reactivation emerges whenever immune surveillance falls below a threshold, regardless of chronological age. Conceptually, age acts as a common proxy for declining surveillance, but transient drops in surveillance can also precipitate off-cycle reactivation in younger hosts without contradicting the model.

## 3 Game-theoretical framework

To formalize the coevolutionary interaction between the host and VZV, we define two strategic options for each player. In our framework, strategies represent abstract choices in the game-theoretical model, whereas phenotypes denote their biological manifestations at the host level (in this manuscript, we use the term phenotype to mean the observable outcomes of VZV infection: a non-lytic latent phenotype, which is clinically silent, and a productive reactivation phenotype, which is clinically manifest as herpes zoster).

### 3.1 Strategies and payoff matrix

To formalize the coevolutionary interaction between the host and VZV, we define two strategic options for each player.

#### 3.1.1 Host strategies

Maintain Immune Surveillance ( S ): The host actively mobilizes its immune system to detect and constrain viral activity.

Allow Immune Suppression ( U ): The host’s immune vigilance is compromised, for example due to aging, immunosenescence, or iatrogenic suppression.

#### 3.1.2 VZV strategies

Latent phenotype ( L ): The virus persists within sensory neurons in a non-lytic state, encompassing a spectrum from true latency (transcriptionally silent) to intermittent animation with limited antigen expression, or subclinical reactivation. All of these manifestations remain clinically silent, though they may help sustain long-term immune surveillance.

Reactivation phenotype ( R ): The virus exits latency and enters a productive replication cycle, characterized by full viral gene expression, DNA replication, and virion production. This process can culminate in clinical herpes zoster (shingles) and onward transmission.

The strategic interplay between host and virus is represented through a payoff matrix that models the relative evolutionary fitness of each strategy combination. Viral payoffs reflect replication potential and persistence, while host payoffs reflect the balance between immune control and the costs of viral activity. The resulting payoff matrix is as follows:

Interpretation of payoff values:

In the first cell (Latency – Surveillance), the virus remains latent under active immune surveillance, maintaining moderate evolutionary fitness by persisting without reactivation. The host obtains maximal benefit not only by suppressing symptomatic reactivation but also by immunologically fortifying the sensory ganglia, thereby enhancing resistance to secondary neuroinvasive pathogens.

In the second cell (Latency – Suppression), latency persists in the absence of immune surveillance with minimal cost to the virus. However, the host fails to retain the added protective advantage conferred by ganglionic immune activation, resulting in a lower net benefit. The evolutionary success of a latent virus fundamentally hinges on the long-term survival and health of its host. A host compromised by increased susceptibility to other debilitating conditions or premature mortality directly reduces the duration of viral latency and, critically, limits future opportunities for reactivation and transmission. Consequently, while the immediate immune cost to the virus may be low in such scenarios, the long-term evolutionary payoff associated with maintaining host viability and propagation potential is significantly diminished.

The third cell (Reactivation – Surveillance) represents a scenario in which viral reactivation attempts are neutralized by the host immune system, preventing any viral gain. Nonetheless, the host suffers immunopathological damage due to inflammation in vulnerable neural tissue.

In the fourth cell (Reactivation – Suppression), the virus reactivates and replicates freely in the absence of immune resistance, achieving maximal viral fitness. The host, however, incurs direct viral damage, and the long-term integrity of the ganglia is compromised due to the lack of immunological defense.

The payoff matrix employs cardinal values, allowing not just a preference ranking but a quantification of evolutionary advantage. This enables a more precise analysis of strategy dominance and the derivation of a Nash equilibrium based on differential fitness payoffs. Within this framework, the equilibrium emerges at two distinct strategic pairings: latency under surveillance and reactivation under suppression. Each reflects an evolutionarily stable state (ESS), where neither the host nor the virus can improve its payoff by unilaterally deviating from the strategy, thus satisfying the criteria of a Nash equilibrium.

### 3.2 Strategic implications and temporal partitioning

Under this formalized structure, when host immune surveillance is robust, VZV latency provides a moderate but stable fitness for both players. Conversely, premature viral reactivation in the presence of strong host immunity results in significant immunological retaliation, leading to viral suppression and a negligible viral payoff. In contrast, viral reactivation during a period of host immune suppression yields a high viral payoff, albeit at the cost of host damage.

This game reveals that there is no single, simultaneous Nash equilibrium that holds true across the host’s entire lifespan. Instead, the payoff matrix supports our hypothesis of a temporally partitioned evolutionarily stable strategy (ESS), in which the optimal strategies for both the host and the virus shift according to the host’s immunological phase.

In youth, when immune surveillance is strong, the host strategy effectively suppresses viral reactivation. Under these conditions, latency becomes the optimal strategy for the virus, as it ensures long-term persistence without triggering immune clearance.

In old age, as immune vigilance wanes due to immunosenescence, the viral payoff from reactivation increases. This shift allows VZV to opportunistically reactivate, leading to successful replication and potentially enabling intergenerational transmission.

Thus, this game-theoretical model elegantly captures a time-dependent strategic balance, where the co-evolutionary dynamics of the host–virus interaction adaptively reconfigure across the host’s lifespan.

### 3.3 Mathematical interpretation

To rigorously formalize the strategic interaction between host immunity and VZV replication, we model their relationship as a two-player evolutionary game with a 2×2 payoff matrix, as shown in **Table 1**. The host can adopt either immune surveillance (Strategy S ) or allow immune suppression (Strategy U ). Concurrently, the virus can choose between latency (Strategy L ) or reactivation (Strategy R ).

**Table 1:** Baseline 2×2 payoff matrix for the host–VZV strategic interaction.

	Host: surveillance ( S )	Host: suppression ( U )
VZV: Latency ( L )	( V : +2, H : +3)	( V : +1, H : +1)
VZV: Reactivation ( R )	( V : 0, H : -2)	( V : +4, H : -1)

The payoffs are defined as follows:

Let $H_{LS}$ , $H_{LU}$ , $H_{RS}$ , $H_{RU}$ represent the host’s payoffs under each of the four possible interactions: (Latency, Surveillance), (Latency, Suppression), (Reactivation, Surveillance), and (Reactivation, Suppression), respectively.

Similarly, $V_{LS}$ , $V_{LU}$ , $V_{RS}$ , $V_{RU}$ ​ denote the corresponding payoffs to the virus under the same strategy combinations.

Let p denote the probability that the host employs immune surveillance, and q the probability that the virus remains latent.

#### 3.3.1 Nash equilibrium conditions

In a mixed-strategy Nash equilibrium, each player must be indifferent between their two available strategies, implying that the expected payoffs for each of their strategies are equal.

For the host, the expected payoff from surveillance must equal that from suppression:


$$q\cdot H_{LS}+\left(1-q\right)\cdot H_{RS}=q\cdot H_{LU}+\left(1-q\right)\cdot H_{RU}$$

Similarly, for the virus to be indifferent between latency and reactivation, its expected payoff from latency must equal that from reactivation:


$$p\cdot V_{LS}+\left(1-p\right)\cdot V_{RS}=p\cdot V_{LU}+\left(1-p\right)\cdot V_{RU}$$

Solving the indifference conditions yields


$$p^{*}=\frac{V_{RU}-V_{RS}}{(V_{LS}-V_{LU})-\left(V_{RS}-V_{RU}\right)},q^{*}=\frac{H_{RU}-H_{RS}}{\left(H_{LS}-H_{LU})-(H_{RS}-H_{RU}\right)}$$

Let $D_{V}=\left(V_{LS}-V_{LU}\right)-\left(V_{RS}-V_{RU}\right)$ and $D_{H}=\left(H_{LS}-H_{LU}\right)-\left(H_{RS}-H_{RU}\right)$ .


$$p^{*}=\frac{V_{RU}-V_{LU}}{D_{V}},q^{*}=\frac{H_{RU}-H_{RS}}{D_{H}}$$

Note that $p^{*}$ depends exclusively on viral payoffs $\left(V_{\cdot \cdot }\right)$ , whereas $q^{*}$ depends exclusively on host payoffs $\left(H_{\cdot \cdot }\right)$ . Under the present payoff configuration $(D_{V},D_{H}>0$ and $V_{LS}>V_{LU},\;H_{LS}>H_{LU}$ ; typically also $V_{LS}\geq V_{RS})$ , an increase in $V_{RU}$ or $V_{RS}$ raises $p^{*}$ , whereas an increase in $V_{LS}$ or $V_{LU}$ lowers $p^{*}$ . By contrast, $q^{*}$ varies only with host payoffs: a decrease in $H_{LS}$ , an increase in $H_{LU}$ or $H_{RU}$ , or a shift of $H_{RS}$ toward more negative values each tends to raise $q^{*}$ (conversely, making $H_{RS}$ less negative lowers $q^{*}$ ).

Hope-Simpson originally proposed the concept of an *immune threshold* as a fixed requirement of host immunity to prevent reactivation (26, 27). However, no specific quantitative value has ever been empirically defined. In our framework, this notion is reformulated as a dynamic equilibrium condition emerging from host–virus interactions. Thus, what appeared in Hope-Simpson’s original formulation as a static threshold should be understood instead as a dynamic balance point that shifts with changes in the payoff matrix. In other words, the immune threshold is not a universal constant but a context-dependent outcome of the evolutionary game between host and virus. A concrete example is provided by immunosenescence: as host immune payoffs decline with age, the equilibrium shifts toward lower values of $q^{*}$ , meaning that surveillance becomes less frequently sustained. This altered balance raises the effective probability of viral reactivation, illustrating how the immune threshold moves dynamically rather than remaining fixed.

#### 3.3.2 Temporally partitioned evolutionarily stable strategies

The concept of an evolutionarily Stable Strategy (ESS), first defined by Maynard Smith and Price (28), and further developed by Maynard Smith (29), posits that a strategy S is an ESS if, when prevalent in a population, no rare mutant strategy M can successfully invade and replace it. Formally, this occurs if E(S,S)>E(M,S) or if E(S,S)=E(M,S) and E(S,M)>E(M,M) where E(X,Y) denotes the payoff to strategy X when interacting with strategy Y .

Within this framework, the dynamics of viral strategies, particularly for viruses like VZV, reveal a limitation of classical ESS when accounting for host physiological changes over time. During early adulthood, when host immune surveillance is robust, latency ( L ) serves as the resident strategy. Under these conditions, a mutant reactivation strategy ( R ) is selected against, as E(L, L)>E(R,L) , satisfying the initial condition for evolutionary stability.

However, the efficacy of the latent strategy is not static. As the host ages, immune competence invariably declines. This physiological shift is mathematically represented by the diminishing negative impact of viral reactivation on the host (i.e., the payoff term $H_{RU}$ becomes less negative), thereby altering the payoff structure to E(L, L)<E(R,L) . This change permits the reactivation strategy to invade and eventually dominate the population. This age-dependent transition underscores a crucial temporal modulation in strategic stability: latency remains evolutionarily stable under strong immune surveillance, but it yields to reactivation as host defenses deteriorate over time.

To formally characterize this temporal dependency, we introduce the concept of a temporally partitioned evolutionarily stable strategy (TP-ESS). A TP-ESS is defined as a strategy whose stability is contingent upon an internal physiological parameter that varies over time.

Let S(t) denote the resident strategy at time t , and M a mutant strategy. The internal host state, such as immune surveillance capacity, is represented by θ(t) , which typically declines with age ( θ(t)↓ ). The payoff to strategy X when interacting with strategy Y , under internal condition θ(t) , is expressed as E(X, Y;θ(t)) .

The conditions for a TP-ESS are then given by:


$$\begin{array}{l} \begin{array}{cc} \exists \;t_{1},\;t_{2}\;such\;that:\{\begin{array}{c} E(L,\;L;\theta \left(t\right)>E\left(R,\;L;\theta \left(t\right)\right),\;t\in \left[0,t_{1}\right]\\ E(R,\;R;\theta \left(t\right)>E\left(L,\;R;\theta \left(t\right)\right),t\geq \;t_{2} \end{array} & \begin{array}{l} \left(Latency\;ESS\;phase\right)\\ \left(Reactivation\;ESS\;phase\right) \end{array} \end{array} \end{array}$$

Here, $\theta \left(t_{1}\right)=\theta_{c}$ denotes a critical immune threshold. Beyond this threshold, the evolutionary advantage shifts from latency to reactivation. This framework integrates time as a third dimension into classical ESS theory, allowing for distinct strategies to maintain stability at different stages of the host’s lifespan. By doing so, it accurately reflects the biological reality of immune decline and the subsequent reoptimization of viral strategies over time, thereby extending classical ESS into a temporally dynamic domain.

#### 3.3.3 Sensitivity of temporally partitioned ESS to payoff parameters

Building on the closed-form expressions for the mixed-strategy probabilities introduced in section 3.3.1—and under the same positivity assumptions on the denominators $D_{V}$ and $D_{H}$ —the equilibrium decouples: $p^{*}$ is determined solely by the viral payoffs, whereas $q^{*}$ is determined solely by the host payoffs.

On the viral side, enlarging the rewards to reactivation—either under host suppression $\left(V_{RU}↑\right)$ or under host surveillance $\left(V_{RS}↑\right)$ —increases $p^{*}$ ; by contrast, enlarging the rewards to latency—under surveillance $\left(V_{LS}↑\right)$ or suppression $\left(V_{LU}↑\right)$ —decreases $p^{*}$ . Intuitively, when reactivation becomes more profitable, a higher frequency of host surveillance is required to keep the virus indifferent between latency and reactivation, which narrows the parameter region in which latency prevails. In our payoff specification, the viral payoff for attempting reactivation when the host maintains surveillance $\left(V_{RS}\right)$ is typically small—often non-positive—so although $p^{*}$ increases with $V_{RS}$ in principle, changes in $V_{RS}$ have limited practical impact on $p^{*}$ under ordinary immune conditions.

On the host side, reducing the payoff for latency under surveillance $\left(H_{LS}↓\right)$ , increasing the payoff for latency under suppression $\left(H_{LU}↑\right)$ or for reactivation under suppression $\left(H_{RU}↑\right)$ , or shifting the payoff for reactivation under surveillance toward more negative values $\left(H_{RS}↓\right)$ each raises $q^{*}$ ; conversely, making $H_{RS}$ less negative lowers $q^{*}$ . A larger $q^{*}$ indicates that the host requires a higher frequency of viral latency to remain indifferent between surveillance and suppression. If the realized latency frequency q falls below this threshold, suppression becomes the host’s best response and conditions tilt toward viral reactivation.

Viewed through this lens, immunosenescence can be represented as a decline in the host payoff for latency under surveillance $\left(H_{LS}↓\right)$ and/or a rise in the host payoff for reactivation under suppression $\left(H_{RU}↑\right)$ —both effects raise $q^{*}$ and broaden the circumstances under which suppression, and thus reactivation, can dominate. Accordingly, the model not only captures the instantaneous strategic configuration between host and virus but also accounts for its evolution across the host lifespan, supporting a time-partitioned coevolutionary equilibrium in the VZV–host relationship.

## 4 The immunosensor hypothesis: a new role for latent VZV

Sensory ganglia, located at the interface between the peripheral and central nervous systems, are uniquely vulnerable because they lack a fully protective blood–brain barrier (BBB) and contain neurons whose axons project directly into the CNS (30, 31). To mitigate this vulnerability, they must deploy local immune defenses that are strong enough to deter pathogens yet restrained enough to prevent immune-mediated neuronal injury. This arrangement can be compared to a fortified gatehouse: just as a narrow entry can be defended by relatively few soldiers, sensory ganglia are protected by a modest contingent of immune cells, sufficient to repel potential invasion while minimizing collateral neuronal injury.

Latency of varicella-zoster virus (VZV) provides a mechanism for such balanced defense. By maintaining low-level antigenic stimulation, latency sustains a modest yet vigilant immune presence within ganglia. VZV persists in neurons that express low levels of MHC class I and virtually no MHC class II. In this context, immune surveillance is orchestrated primarily by CD4^+^ T cells that adopt a tissue-specific memory (TSM) phenotype. Antigen-presenting cells (satellite glia, macrophages, dendritic cells) intermittently process viral gene or protein products—a phenomenon termed *viral animation*—and present them via MHC class II. TSM CD4^+^ cells then secrete cytokines including IFN-γ, IL-17, and TNF-α, which activate NK cells, macrophages, and neutrophils, thereby maintaining a controlled but active immune environment that suppresses viral reactivation.

Although CD4^+^ T cells dominate this response, CD8^+^ T cells are also present in VZV-latently infected ganglia. Human postmortem analyses confirm the presence of VZV-specific CD8^+^ T cells, but their relatively low abundance suggests that their role in maintaining latency is less prominent than in HSV-1, where CD8^+^ infiltration is frequent and functionally robust (32). Neurons express only limited MHC class I, restricting opportunities for direct CD8^+^-mediated cytotoxicity, which could otherwise inflict neuronal injury. Instead, CD8^+^ persistence appears to depend on CD4^+^-derived cytokine support, and their contribution is best understood as complementary rather than central.

Importantly, latency is not a quiescent state but a strategic adaptation that integrates multiple immune components. Intermittent viral animation sustains recurrent antigenic input, reinforcing cell-mediated immunity not only through TSM CD4^+^ and CD8^+^ T lymphocytes but also via NK cells, macrophages, dendritic cells, and γδ T cells. Evidence from other herpesviruses, such as murid herpesvirus-4 priming NK cytotoxicity through granzyme B, supports the broader principle that latency can enhance host immune vigilance rather than function solely as viral concealment (33–37).

This framework reflects an increasingly recognized feedback loop between innate and adaptive immunity (38, 39). TSM CD4^+^ cells within sensory ganglia act as resident sentinels that perpetuate local immune circuits through cytokine secretion, sustaining the activity of innate effectors. In parallel, antibodies from long-lived plasma cells opsonize viral antigens, enabling antibody-dependent cytotoxicity by Fc receptor–bearing innate cells. Thus, CD4^+^-dominated surveillance not only preserves latency but also amplifies non-specific immune readiness at the ganglionic site.

In the absence of latency, sensory ganglia would lose this sentinel function, leaving CNS entry zones more susceptible to invasion by other neurotropic pathogens. The immunosensor hypothesis therefore reframes VZV latency as a mutualistic adaptation: the host tolerates persistent viral genomes in exchange for sustained immune vigilance, while the virus secures its persistence. Rather than a quiescent state, latency represents a strategic balance in which sensory ganglia serve simultaneously as viral reservoirs and immunological outposts, contributing to long-term CNS protection.

## 5 Evolutionary parallels in viral persistence strategies

Our TP-ESS framework builds on these prior modeling approaches (6) by extending beyond descriptive persistence–reactivation dynamics. Specifically, we formalize latency not as an unresolved persistent state but as an immunologically active and temporally adaptive strategy, embedded within a coevolutionary game between host and virus. The concept of temporally partitioned strategies, where distinct life stages or environmental contexts favor different evolutionarily stable strategies (ESS), is a widespread phenomenon across diverse biological systems. This evolutionary precedent lends plausibility to our hypothesis of a time-structured viral strategy in VZV. Here, we highlight several examples showing how phased strategies are stabilized by natural selection.

### 5.1 Viral parallels

More direct and compelling parallels emerge from virology. Several human viruses demonstrate how robust immunity can coexist with persistent infection, often maintained through low-level activity or sequestration in immune-privileged niches.

A more virologically grounded parallel is provided by measles virus (MeV). Even though MeV induces strong and durable neutralizing immunity, its clearance is unusually protracted: viral RNA and protein can persist in lymphoid tissues for months after the resolution of acute infection. This persistence does not result in productive replication but provides a low-level antigenic stimulus that supports immune maturation and lifelong protection, while also predisposing, in rare cases, to late-onset neuropathology such as subacute sclerosing panencephalitis (40, 41). In this respect, MeV offers a useful parallel to VZV, which likewise maintains a long-term balance with the host by combining strong adaptive immunity with intermittent viral gene expression, thereby sustaining immune surveillance without continuous productive infection.

Poliovirus also illustrates this principle. Although acute infection is typically cleared rapidly, viral RNA can be shed from the gastrointestinal tract for extended periods even in the presence of neutralizing antibodies, highlighting that strong immunity does not preclude prolonged persistence (42).

Ebolavirus demonstrates another variant: survivors of acute infection may harbor viral RNA and proteins in immune-privileged compartments such as the eye, central nervous system, or semen for months to years. These reservoirs can occasionally give rise to infectious virus, underscoring how viruses exploit anatomical niches to sustain long-term survival despite systemic immunity (43).

JC virus, a ubiquitous polyomavirus, persists asymptomatically in most adults but can reactivate under immunosuppression to cause progressive multifocal leukoencephalopathy. This model closely parallels VZV, where lifelong latency is normally contained by immune surveillance but reactivation ensues when surveillance falters (44).

Finally, cytomegalovirus (CMV) exemplifies a strategy of lifelong latency punctuated by subclinical reactivations. These episodes continuously stimulate T-cell responses, a phenomenon termed *memory inflation*, ensuring viral persistence even under constant immune pressure (45).

Collectively, these virological examples reveal a unifying theme: persistence is not simply a failure of clearance but an evolved strategy. Viruses can combine strong adaptive immunity with intermittent gene expression, subclinical reactivation, or anatomical sequestration to maintain long-term survival. VZV fits squarely within this broader framework of viral persistence strategies.

### 5.2 Divergent temporal strategies of herpesviruses: HSV by frequency, VZV by coverage

Herpes simplex virus (HSV) exemplifies a high-frequency, low-coverage life-history strategy. Across the host lifespan, HSV undergoes numerous reactivation events that generate recurrent mucocutaneous lesions and facilitate efficient transmission through oral or sexual contact. This strategy creates many short-interval transmission opportunities per host, but its success is tightly linked to individual behavior and constrained by rapid immune containment at epithelial and mucosal barriers (46).

Varicella-zoster virus (VZV), in contrast, represents a low-frequency, high-coverage strategy. Primary infection in childhood results in widespread replication in the respiratory tract and skin, ensuring highly efficient airborne and droplet transmission under conditions of group living. This process achieves near-universal infection within a cohort, after which the virus establishes latency in sensory ganglia. Latency functions as a stable long-term reservoir, while localized immune priming further supports persistence. Reactivation is relatively rare, typically occurring with immunosenescence in later life, and serves primarily as a supplementary transmission route rather than a prerequisite for viral maintenance (10).

From an evolutionary standpoint, this divergence reflects ecological opportunity and host demography. In ancestral human populations, high childhood mortality reduced mean life expectancy, yet individuals surviving early childhood often lived for several further decades (47). Under such conditions, VZV’s reliance on pediatric airborne transmission was sufficient to ensure near-universal carriage, with occasional late-life reactivation acting as a safeguard to reintroduce virus to new susceptibles. HSV, by contrast, capitalized on adolescent and adult behaviors such as kissing and sexual contact, favoring frequent reactivation and recurrent localized shedding as its dominant persistence strategy.

Taken together, these comparisons illustrate how distinct herpesvirus life-history strategies can each be stabilized by natural selection. HSV secures fitness through repeated, behaviorally mediated transmission events, whereas VZV ensures persistence by saturating early cohorts and maintaining lifelong latency with only occasional reactivation. In summary, HSV thrives by frequency, while VZV thrives by coverage.

### 5.3 Niche-based interpretation and evolutionary dynamics

Within the framework of viral ecology, the *true niche* of a virus is defined by the site of productive replication. HSV replicates most efficiently at mucocutaneous surfaces, utilizing sensory ganglia primarily as a refuge for latency. By contrast, VZV establishes sensory ganglia as its permanent home, coupling latency to intermittent animation events that sustain localized immune vigilance (48). This asymmetry helps explain why superinfection exclusion appears attenuated when the two viruses coexist within the same ganglion: HSV behaves as a tolerated guest, whereas VZV assumes the role of resident owner of the neuronal niche (49–51).

This distinction also clarifies the payoff structure when one virus reactivates. If VZV reactivates while HSV remains latent, VZV gains a short-term transmission advantage but destabilizes the shared environment, thereby imposing a net cost on HSV latency. Conversely, if HSV reactivates, it secures an immediate benefit through localized shedding, yet the heightened immune surveillance exposes VZV to indirect costs by undermining its long-term mutualism strategy, despite the limited antigenic overlap between the two viruses. Conceptually, this dynamic resembles a Prisoner’s dilemma: both viruses achieve the highest long-term payoff by maintaining latency, but once one defects through reactivation, the other is pressured toward co-reactivation, culminating in a mutually deleterious outcome that represents the breakdown point of temporal mutualism (52–55).

Finally, the VZV strategy may reflect the imprint of an evolutionary remnant. In ancestral hunter–gatherer societies, humans lived in small, mobile groups with limited and sporadic interpersonal contact. Under such demographic and ecological conditions, encounters with novel viruses and bacteria were relatively rare but often carried high pathogenic risk when they did occur. Within this setting, intermittent animation of VZV latency may have conferred host-side benefits by boosting immune vigilance and thereby enhancing resistance against the invasion of newly encountered pathogens. In modern contexts of improved hygiene and reduced pathogen exposure, however, this protective dimension has largely receded, leaving the pathogenic burden of zoster as its most visible legacy.

## 6 Information- and fitness-based validation of temporal mutualism

Validation of temporal mutualism requires complementary perspectives from information theory and evolutionary fitness modeling.

### 6.1 Entropy-based evaluation of viral animation

Direct measurement of viral markers in human sensory ganglia—such as VLT transcripts, ORF63 expression, or IE63 protein—is not feasible in living subjects. Consequently, studies must rely on host-side proxies of viral animation, with VZV-specific CD4^+^ T-cell responses providing the most practical readout, quantified by IFN-γ ELISPOT for the frequency of responding cells and by intracellular cytokine staining (ICS) for polyfunctionality through simultaneous IFN-γ, IL-2, and TNF-α expression. The live attenuated zoster vaccine (Zostavax), which can establish latency in sensory ganglia, offers a unique model in which such immune responses can be monitored under controlled conditions. Within this framework, Shannon entropy provides a principled means to assess the temporal distribution of reminder signals. A strictly periodic pattern would yield very low entropy, producing little new information and risking immune habituation, whereas a purely random pattern would yield very high entropy, provoking excessive immune activation. The most efficient regime is intermediate entropy, where quasi-stochastic bursts provide reminder signals that sustain CD4^+^ T-cell memory without exhausting host resources. Empirically, this hypothesis can be explored after Zostavax vaccination by analyzing the distribution of inter-event intervals in immune readouts. Latency-associated bursts are expected to fall between the extremes of fixed periodicity and Poisson-like randomness, thereby supporting the view that VZV latency operates within an intermediate-entropy regime that reinforces durable immunity.

### 6.2 Fitness-based validation of temporal mutualism

Beyond information-theoretic analysis, temporal mutualism can also be evaluated through an evolutionary fitness perspective. The central prediction is straightforward: viral lineages with excessively high mutation rates accumulate deleterious changes, destabilize latency, and increase the risk of immune clearance, whereas lineages with very low mutation rates preserve antigenic integrity, maintain latency-associated immune signaling, and ensure long-term persistence. For VZV, which already achieves near-universal transmission in childhood, the marginal benefit of immune escape through frequent mutation is negligible compared with the advantage of maintaining a stable latency program. Evolutionary simulations support this principle: high-mutation variants are progressively outcompeted, while low-mutation variants persist and dominate. Moreover, once established, a low-mutation population resists invasion by higher-mutation competitors, underscoring the selective advantage of genomic stability (56–59). This convergence toward low mutation rates aligns with the observed evolutionary conservation of VZV and supports the hypothesis that latency is not a silent byproduct but a deeply entrenched strategy shaped by natural selection.

## 7 Clinical implications

Accumulating epidemiological evidence indicates that vaccination in adulthood may confer protection against dementia. A recent meta-analysis of over 1.8 million participants reported that vaccination overall was associated with a 35% lower risk of dementia, with protective effects observed across multiple vaccines, including rabies, Tdap, herpes zoster, influenza, hepatitis A, typhoid, and hepatitis B. The benefit was greatest in individuals receiving multiple vaccine types or repeated influenza vaccination, independent of age and sex (60). Pneumococcal vaccination has likewise been linked to up to a 63% reduction in Alzheimer’s disease risk (61). In this context, herpes zoster vaccination, particularly with the recombinant subunit vaccine Shingrix, has been associated with reduced dementia incidence. The most direct explanation is that vaccination suppresses VZV reactivation, thereby preventing recurrent ganglionic and CNS inflammation that may accelerate neurodegenerative processes. This interpretation is consistent with the immunosensor hypothesis: Shingrix induces durable CD4^+^ T-cell responses, stabilizing latency and reducing the risk of CNS invasion by effectively limiting opportunities for VZV reactivation (62–64). An additional possibility is that VZV reactivation may trigger latent HSV-1 in the brain, thereby initiating Alzheimer’s-like pathology; vaccination against VZV could thus indirectly suppress HSV-1 activity. Nevertheless, current evidence suggests that the primary protective effect derives from suppression of VZV itself (65, 66).

Further insights arise from comparison of the Oka vaccine strain with wild-type VZV. The Oka strain establishes latency but shows reduced frequency and amplitude of reactivation, including diminished animation, relative to wild-type virus. Clinically, this attenuation lowers the incidence of herpes zoster among vaccinees, but may also shorten the durability of VZV-specific CD4^+^ T-cell memory and shift the age distribution of zoster toward earlier onset (20, 67–71). From the perspective of temporal mutualism, this constitutes a natural experiment. Wild-type VZV has been evolutionarily optimized to balance persistence with immune surveillance through intermittent animation, thereby reinforcing host CD4^+^ T-cell memory while incurring the pathogenic risk of zoster. In contrast, the attenuated Oka strain reduces zoster risk but may diminish the immune training ordinarily reinforced by wild-type latency and its occasional animation events. Thus, vaccination reshapes an evolved viral strategy: it reduces clinical burden but alters the long-term equilibrium between viral persistence and host immunity.

Additional clinical evidence comes from bone marrow transplantation. In one study (72), subclinical VZV viremia was detected in 19% of bone marrow transplant recipients, and when clinical herpes zoster was included, the overall reactivation rate reached 41%. Before these events, VZV-specific T-cell responses were nearly absent. However, they increased markedly following either subclinical or clinical reactivation. From the TP-ESS perspective, this shows that VZV latency is not a silent state but a strategic program. Intermittent antigen exposure (viral animation) and restricted reactivation retrain and sustain host T-cell immunity. Clinical herpes zoster occurs only in a subset of individuals and can be interpreted as a sacrificial reactivation event, functioning like natural booster vaccinations at the population level. Although detrimental to the affected individual, it promotes viral transmission while reinforcing immune memory for the community. Thus, latency can be understood as a temporally partitioned strategy: childhood ensures widespread transmission, adulthood maintains immune surveillance through subclinical activation, and later-life zoster provides supplementary transmission and immune boosting.

If VZV were to reactivate simultaneously across many ganglia, it might yield more virions in the short term. Yet this would come at the cost of severe inflammation, neuronal injury, and strong immune clearance of the remaining latent virus in other ganglia. Such outcomes would jeopardize long-term persistence and disrupt temporal mutualism. In contrast, restricted reactivation in a limited number of ganglia minimizes host damage while still providing sufficient antigenic stimulation to sustain T-cell memory. These occasional zoster episodes can therefore be interpreted as sacrificial reactivation events that help maintain collective immunity while allowing the virus to persist in equilibrium with its host.

## 8 Conclusion

It is now widely acknowledged that VZV latency is not simply a quiescent state, but a dynamic process shaped by continuous virus–host interactions. This broader understanding provides the foundation for considering latency not as an incidental outcome, but as an integral part of the viral life cycle. In this framework, we propose that VZV latency can be interpreted as a temporally partitioned evolutionarily stable strategy (TP-ESS).

Within this framework, VZV’s triphasic life cycle can be viewed as an adaptive equilibrium shaped by natural selection across long evolutionary timescales. During childhood, lytic replication and airborne transmission exploit the immunological naivety and close contact of pediatric hosts. Thereafter, the virus establishes latency within sensory ganglia—immune-privileged sites that ensure durable persistence while functioning as an immunosensor to reinforce host immune surveillance. With advancing age and immunosenescence, reactivation occurs opportunistically, initiating a final wave of transmission later in life. Each phase thus represents a temporally structured solution to shifting host immune environments, maintained not by viral intent but by natural selection.

This perspective positions latency as a coevolved and biologically regulated mutualism, in which host and virus remain in dynamic balance. By interpreting VZV latency through evolutionary game theory and ESS modeling, we gain explanatory insight into its paradoxical features and predictive leverage for understanding how VZV dynamics will unfold under changing demographic and immunological conditions. Ultimately, VZV latency should be recognized as an evolutionarily optimized strategy—one that secures persistence, modulates host immunity, and exemplifies how pathogens and hosts co-adapt within shared ecological and temporal constraints.

## Data Availability

The original contributions presented in the study are included in the article/supplementary material, further inquiries can be directed to the corresponding author/s.
